# A Computational Model of Torque Generation: Neural, Contractile, Metabolic and Musculoskeletal Components

**DOI:** 10.1371/journal.pone.0056013

**Published:** 2013-02-06

**Authors:** Damien M. Callahan, Brian R. Umberger, Jane A. Kent-Braun

**Affiliations:** Department of Kinesiology, University of Massachusetts Amherst, Amherst, Massachusetts, United States of America; Cinvestav-IPN, Mexico

## Abstract

The pathway of voluntary joint torque production includes motor neuron recruitment and rate-coding, sarcolemmal depolarization and calcium release by the sarcoplasmic reticulum, force generation by motor proteins within skeletal muscle, and force transmission by tendon across the joint. The direct source of energetic support for this process is ATP hydrolysis. It is possible to examine portions of this physiologic pathway using various in vivo and in vitro techniques, but an integrated view of the multiple processes that ultimately impact joint torque remains elusive. To address this gap, we present a comprehensive computational model of the combined neuromuscular and musculoskeletal systems that includes novel components related to intracellular bioenergetics function. Components representing excitatory drive, muscle activation, force generation, metabolic perturbations, and torque production during voluntary human ankle dorsiflexion were constructed, using a combination of experimentally-derived data and literature values. Simulation results were validated by comparison with torque and metabolic data obtained in vivo. The model successfully predicted peak and submaximal voluntary and electrically-elicited torque output, and accurately simulated the metabolic perturbations associated with voluntary contractions. This novel, comprehensive model could be used to better understand impact of global effectors such as age and disease on various components of the neuromuscular system, and ultimately, voluntary torque output.

## Introduction

**Figure 1:**
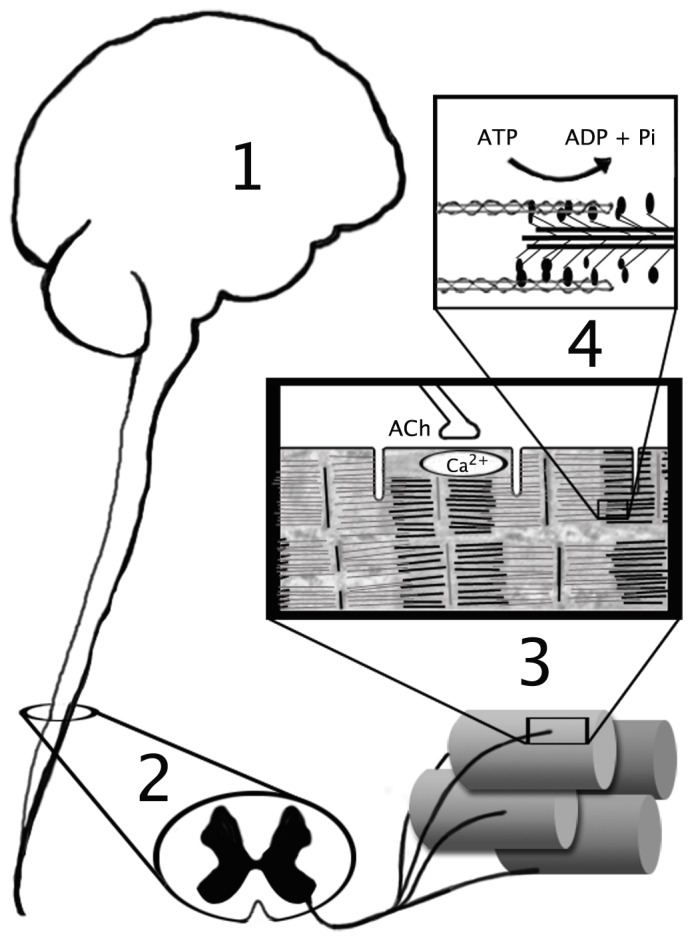
Pathway of Voluntary Torque Production. Physiological events necessary for voluntary torque production are modeled by components in the present computational model. 1) Excitation in the motor cortex 2) α motor neuron activation, 3) sarcolemmal depolarization, calcium release, 4) cross-bridge formation and muscular force development. Reproduced from: Kent-Braun J.A., Fitts R.H., Christie A. “Skeletal Muscle Fatigue” In: Comprehensive Physiology, Wiley-Blackwell, 10.1002/cphy.c110029, 2(2):997–1044, 2012.

Although muscle cross sectional area is the greatest determinant of maximal isometric joint torque in humans [1], only about two-thirds of maximal torque is accounted for by muscle size. In fact, there are examples in the literature of considerable variation in maximal voluntary torque per unit area [N·m·cm^−2^]) [2], also termed “specific strength” [1]. The generation of voluntary torque, illustrated in Figure 1, begins with neural excitation in the motor cortex, which produces propagation of excitatory potentials down the cortico-spinal tracks to the α motor neurons. These motor neurons innervate muscle cells, causing depolarization of the sarcolemma and release of Ca^2+^ from the sarcoplasmic reticulum. In this way, cross-bridge cycling is initiated and force is produced, ultimately leading to torque generation about a joint. Clearly, variation in any of the processes along this pathway (Figure 1) could result in alterations in joint torque. Variations may include changes in motor unit discharge rates, excitation-contraction coupling, muscle fiber contractile force, or the intracellular metabolic milieu. The complex interrelationships among the physiological systems that govern these processes further impact their combined function.

The interrelated nature of the physiological processes involved in the generation of voluntary joint torque is difficult to discern in vivo. While in vitro experimentation can provide explicit details about isolated systems, and in vivo studies typically describe the combined function of multiple systems, each of these approaches is limited in addressing the coordinated events that lead to the development of voluntary joint torque. Modeling and simulation techniques may be used in a complementary fashion with in vitro and in vivo methods to gain unique insights that are not possible using experimentation alone [3]–[7]. Since the pioneering work of A.V. Hill [8] and A.F. Huxley [9], computational models have been proposed to explain a wide variety of functions within the neuromuscular system, including the contractile dynamics of skeletal muscle [10], perfusion [4], neuromuscular activation [11] and fatigue during repeated activations [12]. However, these examples, and most others, focus on a single aspect of a larger system containing many interrelated components.

While such a unitary approach to modeling complicated systems is justified in many cases, a more comprehensive model allows simultaneous inquiry of multiple physiological events associated with the voluntary production of joint torque. A model composed of multiple components of the neuromuscular and musculoskeletal systems might improve our ability to discriminate the relative influence of these components on voluntary joint torque. Critically, it would provide the opportunity to investigate how the relative influence of these components might change in response to global effectors such as age, disuse or disease. Models have been developed that simulate motor neuron recruitment [11], and the spatial distribution of muscle fibers they innervate [4]; depolarization of the sarcolemmal [13]; calcium kinetics [7], [14]; acto-myosin binding kinetics [6], [15], [16]; control of oxidative phosphorylation by ADP [17]; and joint torque [18], [19]; However, rarely have multiple physiological components been included in the same model [12].

The goal of the present study was to develop and evaluate a comprehensive model of neural activation, contractile dynamics, and metabolic perturbation. The approach combined previously-validated models of voluntary activation [11], [20], force development [18], [21], [22] and torque generation [19] with a novel set of model components that predict metabolic perturbation as a consequence of muscle activation. This is the first model to incorporate neural, contractile, bioenergetic, and architectural features of the neuromuscular and musculoskeletal systems for the purpose of simulating human torque production. Incorporation of these features allowed the model to simulate the response of multiple components of the neuromuscular system to the challenge of producing various levels of voluntary joint torque.

## Materials and Methods

### Ethics Statement

Written informed consent, approved by the University of Massachusetts Institutional Review Board, was obtained for all participants prior to their participation. All measures were performed in accordance with the Declaration of Helsinki and this study was approved by the University of Massachusetts institutional review board.

### Approach and Source Data

**Figure 2:**
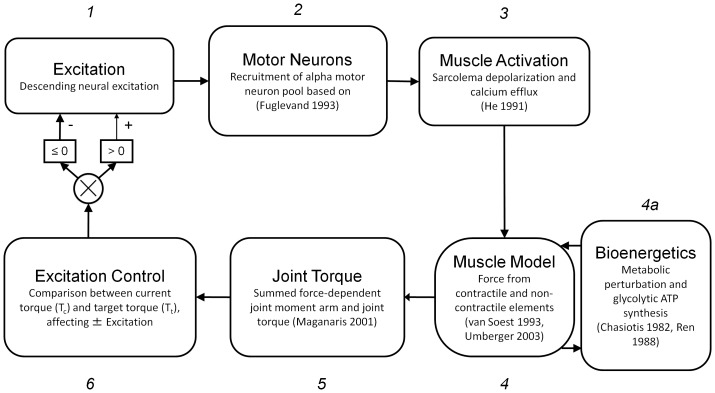
Computational Approach. The model runs using a forward-integration routine to calculate each model step (1–6) at each time point for the duration of the simulation. Primary literature sources pertinent to model functions are listed with each step.

Our approach to an integrated, comprehensive model of neuromuscular function followed the general scheme outlined in Figure 2. Steps in the theoretical model formulation were meant to correspond to the physical pathway of voluntary torque production illustrated in Figure 1. Considerable effort was made to base model parameters on values in published studies of human muscle function from our laboratory [23], [24], and elsewhere in the literature [4], [19], [25], as well as experimental measures available from current and ongoing studies in the Department of Kinesiology at the University of Massachusetts Amherst [26]. Model parameters were based on measures obtained from 8 healthy men (21–35 years), as well as from human studies reported in the literature [11], [27]. The participants underwent ultrasound measures of the anterior shank and magnetic resonance imaging (MRI) of the lower leg to provide anatomical data for formulation of the musculoskeletal component of the model. To formulate the metabolic perturbation component of the model, the participants also underwent metabolic testing using non-invasive, ^31^P magnetic resonance spectroscopy (MRS) [26].

### Computational Overview

Because the model integrates the output from multiple components intended to represent stages in the pathway of torque production (Figure 1), they are presented here in the same sequence that they were computed in the forward dynamics simulations (Figure 2). All simulations for model formulation and analysis were performed using Matlab software (MathWorks, Natick MA). Briefly, a single parameter representing voluntary excitation (Step 1) of the spinal cord initiated the model by serving as the input for a pool of motor neurons (MN). The MN pool responded to the excitation signal according to a recruitment scheme originally proposed by Fuglevand et al [11] (Step 2). A first-order model of activation, representing sarcolemmal depolarization and intracellular calcium release (Step 3), generated a set of signals that served as the inputs to a pool of 120 first-order, Hill-type [18] muscle models (Step 4). Because the output of each MN acted on a corresponding muscle model, this approach effectively simulated the organization and behavior of a motor unit (MU) in vivo [28]. The linear sum of forces produced by all muscle models was then used as the input for a musculoskeletal model of the ankle joint, to predict current joint torque (T*_c_*) at the ankle (Step 5). Finally, T*_c_* was compared with a target torque (T*_t_*) for each time point *t* which allowed the model to adjust excitation (*S*, Step 1) such that the difference between T*_c_* and T*_t_* was minimized. Calculations were performed for time steps of 0.001s and repeated for each MU before advancing the time step. Between each time step, the system of equations associated with the output of each MU was integrated forward in time using the ‘ode45’ differential equation solver in Matlab. Details regarding the major components of the model are detailed below and in appendices A and B.

#### Step 1. Excitation

The initial step for the model represents excitation which begins at the motor cortex and descends through corticospinal tracts to α MNs in the spinal cord. Input from the central nervous system to pools of α MNs is physiologically complex and regulates many aspects of coordinated, voluntary muscle activation. Because of its complicated nature and incomplete definition in the literature, no attempt is made for *S* to directly reflect physical events in the process of cortical excitation. Instead, an approach similar to that employed by Xia et al [29] was used whereby the model adjusts *S* at each time step to minimize the difference (T*_diff_*) between current modeled torque (T*_c_*) and target torque (T*_t_*) with respect to peak torque-generating capacity:
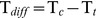
(1)in the case where T*_diff_* is <0,
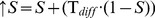
(2)and in the case where T*_diff_* is >0,
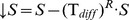
(3)


This simple control algorithm uses the value *R* = 0.7 to minimize unintended relaxation characteristics while maintaining predictions for activation and relaxation that were consistent with in vivo observation. An estimate of peak torque-generating capacity was established by multiplying the sum of peak force-generating capacity for each muscle model by the maximum possible moment arm. This estimate is not necessarily the same as actual peak torque-generating capacity, which is also subject to other model elements (FR, activation, contractile element length and velocity, variable moment arm length based on stretch of the extensor retinaculum). However, it was effective for moderating changes in activation that resembled in vivo experimental observations.

#### Step 2. Motor Neuron Pool

The pool of 120 motor neurons responded to *S* according to procedures described by Fuglevand et al [11]. The recruitment thresholds (RT) for the pool of MNs were distributed such that many MNs had low RT while relatively few had high RT. The distribution of RTs is described by the equation:
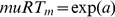
(4)where *muRT_m_* is the RT of MN(m) and 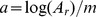
_;_ *A_r_* = 30 is the desired range (fold difference) for *muRT_m_*. A 30-fold range of RT is consistent with the broad variation in recruitment thresholds observed experimentally [28]. Each MN is assigned a minimum firing rate (MFR) of 8 Hz [25], [28]. Although it is possible that MFR could vary between MNs in direct proportion to RT [30], empirical studies performed in humans during voluntary contractions suggest that MFR is constant across MNs [31], [32].

Once a MN's threshold for excitation was surpassed, a single linear function described the relationship between excitation and FR:
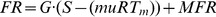
(5)where *G* is a gain function affecting the magnitude of increasing FR and S is the current level of excitation. FR increased according to this function until the given MNs achieved a pre-determined peak firing rate. The peak firing rate for each MN is directly proportional to its RT within the relatively narrow range of 10 Hz [33]. The fastest MNs fired at 56 Hz [25]. Each “pulse” delivered by a given MN model served as the input to a model of muscle activation, described in the next section.

#### Step 3. Muscle Activation

Because the kinetics of the Ca^2+^ transient are significantly slower than those of the depolarization event [34], [35] and precise measurement of the Ca^2+^ transient has not been performed in human skeletal muscle, no effort is made to distinguish the two events in the present model. The combined steps of post-synaptic muscle activation were modeled similarly to the approach used by He et al [21]:
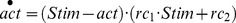
(6)

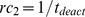
(7)

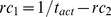
(8)where *t_act_* has values between 0.039–0.060 depending on MN assignment (*m*), and represents the activation time constant. Deactivation time constants are defined by *t_deact_* and have values between 0.064–0.092. These values were based on Umberger and colleagues [22] and modified slightly for the current application where individual MUs were represented. Specifically, values were altered to allow for a range of activation and deactivation kinetics within the MN pool, and co-varied with contractile kinetics (detailed later) to produced rates of force development and relaxation that were physiologically realistic. The value of *Stim* was set to either 0 or 1 and meant to represent the activity of the sarcoplasmic reticulum either releasing or resequesting Ca^2+^ in response to sarcolemmal depolarization. For the first 0.023-s that each MN was activated, the value *Stim* associated with that MN was assigned a value of 1, and 0 thereafter. The counter incremented through the duration of the current interpulse interval (IPI = FR^−1^), after which point the counter was reset to 0, and the process continued until the MN was no longer active. A more detailed description of this process can be found in Appendix S1. This activation strategy yields activation kinetics consistent with the time course of experimentally-observed Ca^2+^ transients (20–30 ms) [36] and allowed complete summation of the Ca^2+^ transient for the MN with the lowest MFR during maximal stimulation.

#### Step 4. Muscle Models

A detailed list of equations describing the behavior of the muscle and metabolic perturbation models can be found in Appendix S1. The text that follows is a general description of the model formulation procedures.

The activation signal from step 3 was input to a standard Hill muscle model that included contractile (CE) and series elastic (SEE) elements [18]. In keeping with the control structure of the model, 120 independent muscle models correspond in a 1∶1 fashion with 120 MNs. This coordination was intended to reproduce the physiological recruitment of MUs. Peak force-generating capacity of each muscle model (*fmax*) was coordinated with *muRT* such that the unit with the lowest RT had the lowest force. Forces were distributed across 120 MNs through a 100-fold range. The sum of *fmax* from all muscle models was 1433.4 N, a value derived from an optimization routine that determined specific tension from the study population [26]. Total myotendonus muscle length was held constant as all modeled contractions were isometric, but CE and SEE length were free to change and behaved according to the equations in Appendix S1. All muscle models included components for eccentric and concentric force development, pennation angle, velocity and length (Equations 13–20 in Appendix S1).

**Figure 3:**
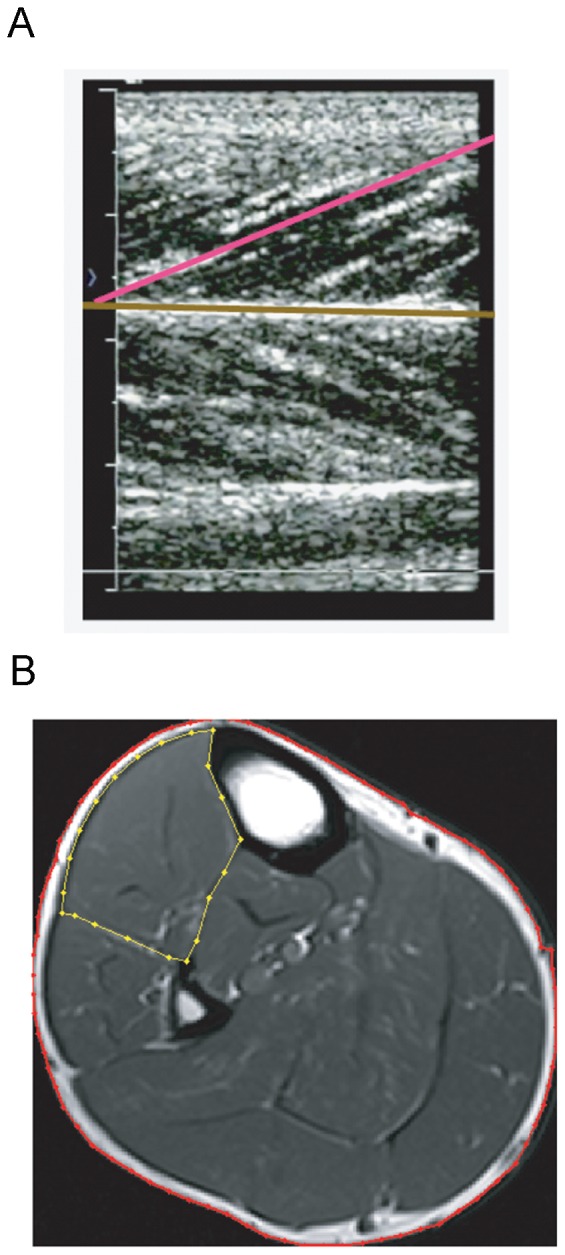
*In vivo* Muscle Imaging. **A:** Ultrasound image of the anterior compartment of the lower limb. The horizontal line matches the orientation of the apponeurosis of the tibialis anterior muscle, and the angled line matches the pennation angle of visible fascicles under the ultrasound probe. **B**: Magnetic Resonance Image (MRI) in the Axial Plane. The dotted line represents the region of interest (ROI) for subsequent analysis of anterior compartment muscle size.

The modeled change in CE and SEE length, along with resulting changes in pennation angle, were based on in vivo ultrasound measures obtained from the study participants. Briefly, ultrasound imaging (Acuson 128XP real-time ultrasonic scanner with linear-array probe, Siemens, Munich Germany) was used to measure the tibialis anterior muscle and tendon while subjects were seated in an isokinetic dynamometer (Biodex, Inc., Shirley New York USA). The ankle was fixed with the foot at 105° relative to the tibia, and subjects performed a torque-tracking task by matching their effort to visual feedback. The subjects steadily increased dorsiflexion torque from rest to maximum voluntary isometric contraction (MVC) over a period of 30-s. During this time, fascicle pennation angle (Figure 3A) and stretch of the tibialis anterior tendon was recorded on videocassette for subsequent analysis using custom-written Matlab software [37].

#### Step 4a. Metabolic Perturbation

The model used current activation level to predict changes in the concentration of intracellular metabolites related to the production of adenosine triphosphate (ATP). The metabolic perturbations associated with active dorsiflexion were modeled after measures of phosphorus-containing metabolites and pH, as described elsewhere [23], [38]. Briefly, ^31^P MRS was used to measure the concentrations of phosphocreatine (PCr) and inorganic phosphate (Pi) using a 3·5 cm surface coil, centered over the tibialis anterior muscle (∼8.4 cm^3^ sample volume) in a 4.0 tesla superconducting magnet (Bruker Biospin, Rheinstetten, Germany). The recovery of PCr following a 12-s MVC was used to calculate the in vivo capacity for oxidative phosphorylation [23], [39] while the chemical shift between Pi and PCr was used to calculate intracellular pH [40], ATP synthesis rates by the creatine kinase reaction, non-oxidative glycolysis and oxidative phosphorylation were estimated from changes in [PCr], [Pi], and pH during contractions at a range of intensities (20%, 50%, and 100% MVC) [23].

Metabolite concentrations were determined every 4 s during contraction and recovery, based on line-fits of each peak using NUTS software (Acorn NMR, Livermore CA). Using these metabolite data, model functions were created to reflect the cost of force production (See Appendix S2) and the subsequent change in cytosolic pH across a range of activation levels (See Appendix S1). The rate of PCr depletion during contractions at 20, 50 and 100% MVC was best fit linearly. The slopes of these lines were used to define the relationship between activation level and the rate of the appearance of Pi (equations 21 and 22 in Appendix S1). This procedure is possible because a constant phosphate pool ([Pi] + [PCr] = 42.5 mM) and ATP concentration ([ATP] = 8.2 mM) can be assumed under these experimental conditions [41], [42]. A three-parameter exponential decay was fit to the data using SigmaPlot software (Systat Software Inc. San Jose, CA) to derive coefficients used to formulate rates of Pi accumulation during activation (equation 1 in Appendix S2).

Intracellular pH was calculated for each muscle model, based on rates of change in [Pi], buffering capacity, and protons produced from the conversion of pyruvate to lactate (H^+^). Glycolytic ATP production rates were estimated from the Michaelis-Menten relationship between Pi and glycogen phosphorylase, where K*_m_* was assumed to be 18.94 mM [43], [44], and the maximum rate of non-oxidative glycolysis was distributed exponentially per *MN* over a 4-fold range (0.48 to 1.92 mM s^−1^, see details in Appendix S1). This range was chosen to reflect rates of glycolysis observed in vivo [45], [46] and resulted in an average across muscle models, weighted to *fmax* (a direct correlate of muscle volume), of 1.5 mM ATP•s^−1^. The rate of H^+^ produced by non-oxidative glycolysis was equal to two-thirds the observed rate of glycolysis, which reflects the rate of proton production for each ATP produced through glycolysis. In vivo, the observation of cytosolic H^+^ production from non-oxidative glycolysis is offset to some extent by the protons consumed in the creatine kinase reaction (net breakdown of PCr). The latter was calculated from the product of a proton stoichiometry coefficient (*θ*) and the rate of Pi accumulation (equivalent to PCr breakdown). The net change in [H^+^] was divided by the current buffering capacity and used to calculate the pH at each time point (equations 29 and 30 in Appendix S1). Finally, the portion of inorganic phosphate in diprotonated form [H_2_PO_4_^−^] was calculated based on current pH and [Pi] (equation 31 in Appendix S1).

#### Step 5. Musculoskeletal Model

The musculoskeletal model was parameterized with data from the group of 8 young men. Muscle architecture measures were performed using ultrasound and magnetic resonance imaging (MRI; 3.0-Tesla MRI system; Siemens, Munich, Germany). To measure muscle volume, serial images (T_1_-weighted spin echo axial images; 4 mm slice thickness, 210 mm field of view, 512×512 matrix) were collected along the total shank length. Custom-written Matlab software was used to first identify a region of interest (ROI) representing the tibialis anterior muscle, and then partition the pixels populating this ROI into contractile and non-contractile tissue based on signal intensity [47]. A sample MRI slice with defined ROI is shown in Figure 3B. Total muscle volume (m^3^) was determined by integrating over the fat-free muscle cross-sectional areas along the length of the muscle.

The forces generated by all simulated motor units were summed linearly to predict force at the tendon (*F_t_*).
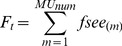
(9)Consistent with in vivo observations [48]–[50], we modeled force-sensitive changes in muscle moment arm length. Force in the dorsiflexor muscles causes the extensor retinaculum to stretch, allowing anterior displacement of the tendon. Thus, muscle moment arm is lengthened as force increases [19]. In our model, current moment arm length (*L_ma_*) was dependent on force *F_t_* such that greater *F_t_* resulted in a larger *L_ma_*.
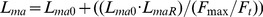
(10)where *L_ma_*_0_ is the moment arm length at rest, and *L_maR_* (0.249) is the relative range of extension past resting length of the moment arm (0.027 m). Increased moment arm above resting values are scaled by the ratio of current force (*F_t_*) to the highest possible force (*F*_max_). The range of moment arm values were based on experimental observation of a single participant using the MRI collection procedures described above. These values were in good agreement with results from Maganaris et al [19]. T_c_ was calculated at the final step.
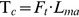
(11)This value was then compared with T*_t_* to obtain T*_diff_* (equation 1).

### Simulation and Evaluation Procedures

Equations describing the behavior of each modeled component were run for each MU at each time step (t), for a range of simulated conditions. As an initial test of the validity of model predictions concerning excitation and contractile dynamics, a series of simulated torque-frequency curves were generated. Briefly, a train of stimuli delivered at a constant frequency was simulated by setting *S* = 1 for the first 0.023 s of each IPI. This procedure was performed across a range of simulated stimulation frequencies. In this paradigm, predicted torque depended on the combined response of many model components to step input changes in *S* as described, and was not parameterized as a discrete function of the model. As a result, the activation and contractile kinetics of the model, as well as musculoskeletal (*L_ma_* and SEE stiffness) components were evaluated simultaneously. Simulated torque was compared with literature values [27] to evaluate the effectiveness of these components in predicting torque-frequency relationships observed in vivo.

Next, a range of voluntary contraction intensities were simulated, for comparison with the experimental data used to parameterize the model as well as literature values. Under these conditions, T*_t_* was set to increase from zero, 1 s into the simulation and remain at 110%, 50%, and 20% of predicted maximal T*_c_* until second 13 of the simulation, thus simulating a 12-s contraction. The T*_t_* value for the maximal stimulation condition was set in excess of 100% to ensure that muscle activation was maximal. Model performance was controlled by auto-regulation of *S* according to equations 1, 2 and 3. Simulated torque, [Pi], [pH] and [H_2_PO_4_^−^] were compared with experimental and literature values and considered valid if the root mean squared difference between them was within one standard deviation of the experimental value in question.

## Results

### Torque – Frequency

**Figure 4:**
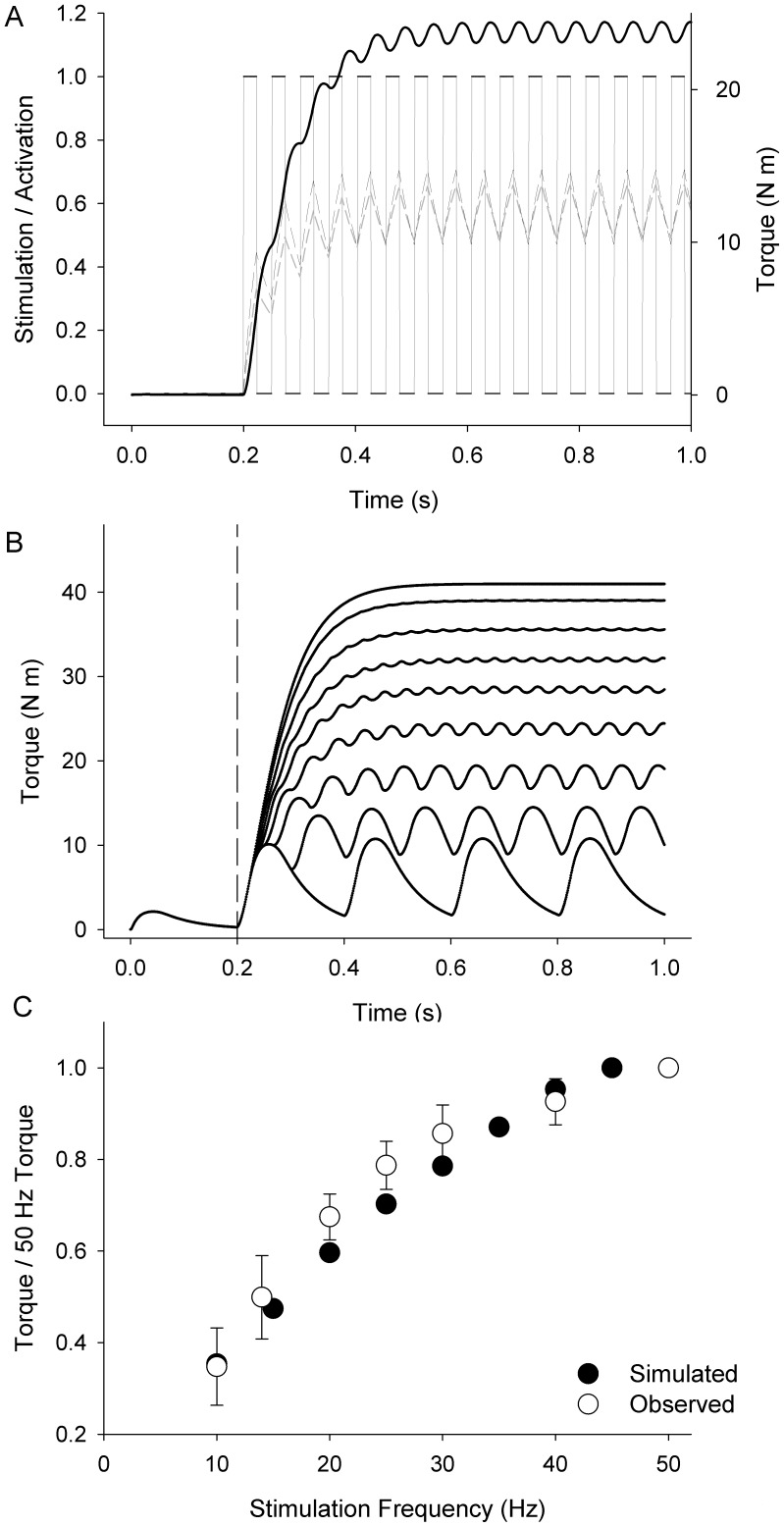
Simulated Torque-Frequency Relationship. **A:** Simulated stimulation protocol was a square wave with pre-determined frequency (20 Hz). Simulated activation responses for the 1^st^ and 60^th^ motor unit are plotted in dark and light grey dashed lines respectively. Total simulated torque for the combined model is plotted in black. **B:** Simulated torque traces in response to stimulation at a range of frequencies (10, 15, 20, 25, 30, 35, 40, and 45 Hz). Stimulation for each simulation began at 0.2 s. **C:** Comparison between simulated and experimental torque output in response to stimulation at frequencies between 10 and 50 Hz. Simulated torque values (closed symbols) were typically within one standard deviation of mean experimental values (open symbols ± SD).

The recruitment and activation values produced by the model agreed well with experimental data. Figure 4A shows model results for neuromuscular stimulation at 20 Hz. The model exhibited pulsatile activation kinetics and wave-summation behavior of torque similar to that observed in vivo. Figure 4B illustrates the simulated torque response to a range of stimulation frequencies between 10 and 50 Hz. The peak torque at each frequency, predicted by the model, is compared with experimental data our laboratory [27] in Figure 4C. Again, the results from the model agreed well with in vivo torque production at all frequencies. The mean squared difference between simulated and observed torque was 5.3% (between 10 and 45 Hz) with a maximal difference of 7.1% at 30 Hz.

### Maximum Voluntary Contraction

**Figure 5:**
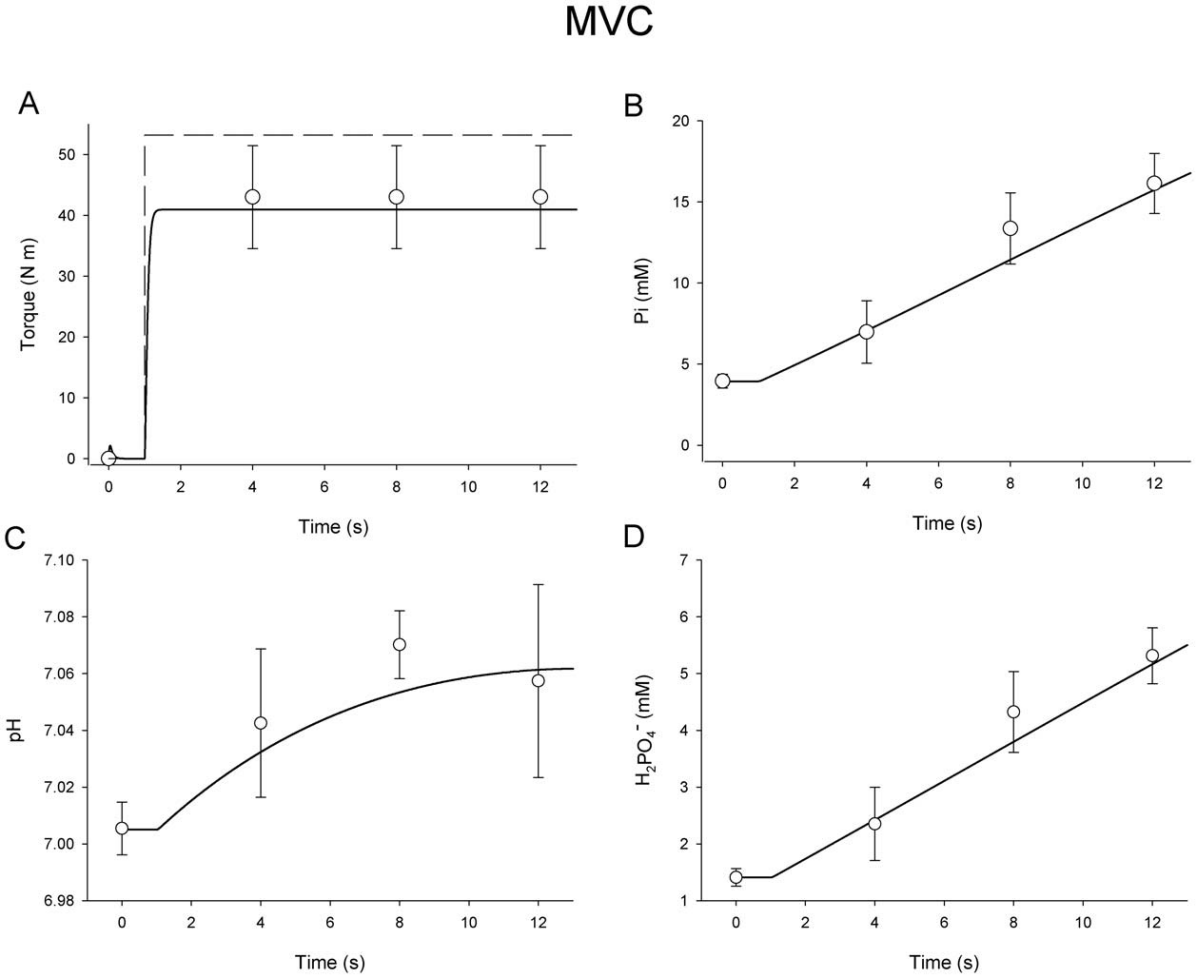
Simulated Maximum Voluntary Contraction. **A:** Simulated torque during 12-s MVC in thin black line with experimental data (open symbols ± SD). Blue line is the target torque (T*_t_*) for the model to approximate. It was set to increase from zero to 110% of expected peak torque output to ensure full activation at t = 1 s. **B:** Simulated (black line) and experimental (open symbols ± SD) inorganic phosphate concentration (mM) during 12-s maximum voluntary contraction. **C:** Simulated (black line) and experimental (open symbols ± SD) pH during 12-s maximum voluntary contraction. **D:** Simulated (black line) and experimental (open symbols ± SD) H_2_PO_4-_ during 12-s maximum voluntary contraction.

During a simulated MVC, T*_t_* was set to 110% of expected peak torque output to promote full excitation in the model (dashed line, Figure 5A). The model (Figure 5A) predicted peak torque within 5.0% of measured torque in our study group of young men. Similar to the in vivo results, the model achieved ∼97% of peak torque within 250 ms. Changes in intracellular [Pi], pH, and [H_2_PO_4_^−^] during the 12-s MVC are compared with in vivo data in Figure 5B–D. Predicted output from the model was within one standard deviation of in vivo measures for all but one time point (pH, 8-s).

### Submaximal Contractions

**Figure 6:**
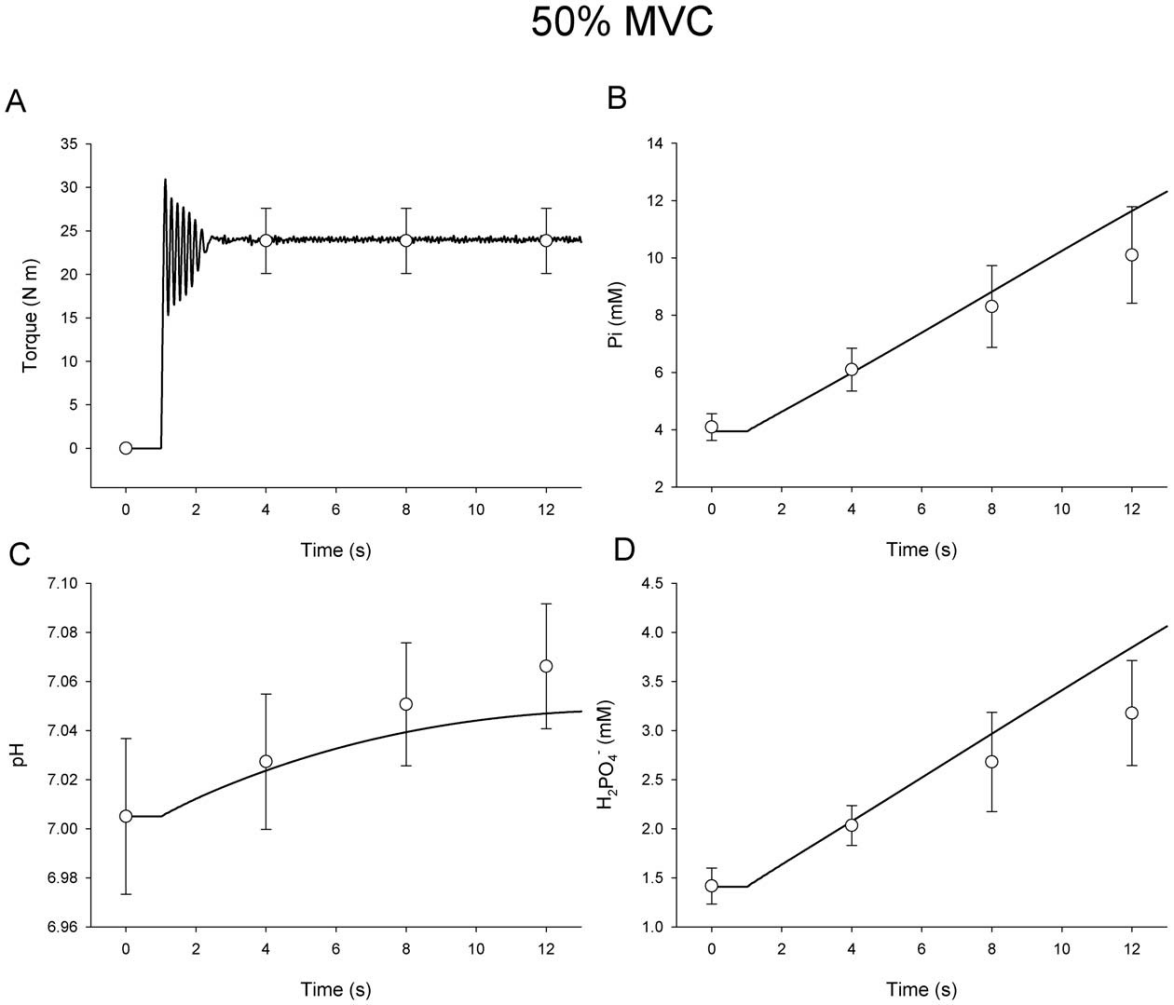
Simulated Submaximal Voluntary Contraction at 50% MVC. Simulated response of Torque (**A**) inorganic phosphate (**B**), pH (**C**), and H_2_PO_4-_ (**D**) during 12-s contraction (black line). In vivo data (open symbols ± SD) are shown for comparison.

**Figure 7:**
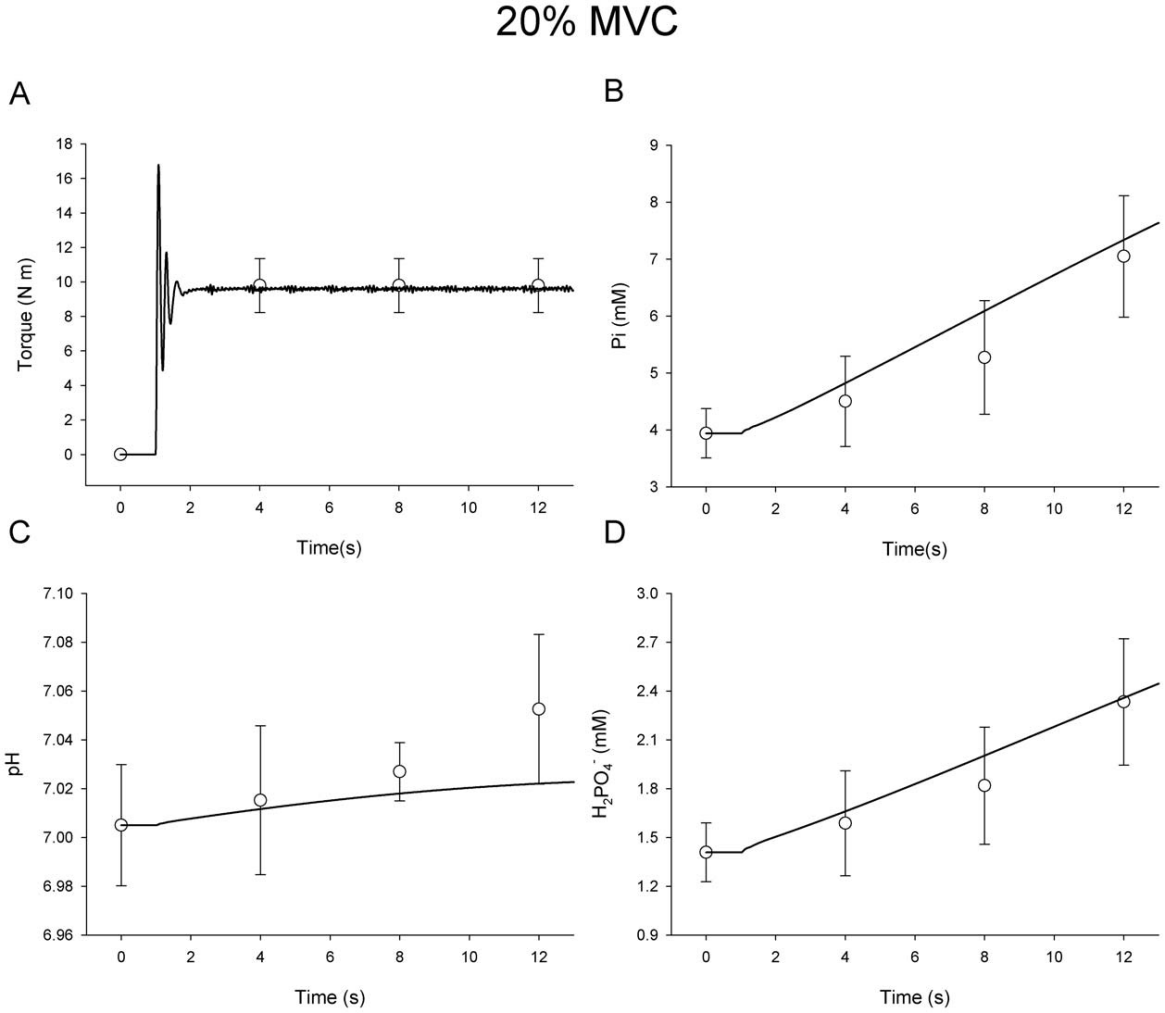
Simulated Submaximal Voluntary Contraction at 20% MVC. Simulated response of torque (**A**) inorganic phosphate (**B**), pH (**C**), and H_2_PO_4-_ (**D**) during 12-s contraction (black line). In vivo data (open symbols ± SD) are shown for comparison.

To investigate the accuracy with which the model matched T*_c_* with T*_t_* during submaximal activations, T*_t_* was set to 50% and 20% of predicted maximum torque capacity. All other aspects of submaximal simulations were identical to MVC simulations. Results from these simulations are shown in Figures 6A and 7A for contractions at 50% and 20% of MVC respectively (note difference in y-axis scales). Torque predicted by the model was within 2.1% of the experimental means for the middle 90% of contraction time in both submaximal simulations. The large amplitude oscillations of T*_c_* about T*_t_* during approximately the first second of torque production (Figures 6A and 7A) reflect the limited resolution of the controller upon a large and rapid change in *S*. While in principle it would be possible to add damping to lessen the oscillations upon activation of the model, torque overshoot followed by an overcorrection is actually a common observation in empirical torque data. Moreover, high-gain functions were necessary to predict accurate metabolic characteristics during deactivation. Thus, the present controller represented a reasonable compromise for simulating both activation and deactivation. In contrast to the initial oscillations observed upon activation of the model, the small amplitude fluctuations of T*_c_* during the remaining 11 seconds of these simulations reflected variations in torque production associated with recruitment and rate coding in the model, and mirrored torque variability often observed in submaximal experimental conditions (c.f. Figure 1a in Yoshitake et al. [51]).

Predicted [Pi], pH, and [H_2_PO_4_^−^] are shown along with in vivo measures during the 12-s contraction at 50% MVC in Figure 6 B, C, and D, respectively. Figure 7 illustrates the same variables during the 12-s contraction at 20% MVC. For [Pi] (Figures 6B and 7B), agreement with experimental data was good, and within the expected physiologic range. The mean squared error for [Pi] was greatest at 12-s in the 50% MVC simulation at 9.8% above in vivo measures. The average error for [Pi] was 6.9% for the duration of the simulated 12-s 50% MVC contraction. There was excellent agreement with predictions of [H_2_PO_4_^−^] during the 50% contraction, with an average error of 2.0% and a peak of error of 4.0% at 12-s. Estimates of [Pi] were slightly elevated during the simulation of a 20% MVC contraction when compared with in vivo data. On average, estimates of [Pi] were 9.7% greater than in vivo measurement with a peak difference of 19.7% at 8-s. This variance was within the standard deviation of the measured mean at all time points, however. Note that the alkalosis normally observed during a brief contraction was slightly under-predicted by the 12-s time-point in the 20% MVC simulation (Figures 7C). Average [H^+^] during this simulation was within 2.3% of measured values with a peak difference of 7.0% at 12-s. Predictions for [H_2_PO_4_^−^] were also reasonable, given physiologic variability, with an average error of 6.5% and a peak difference of 13.9% at 8 s.

## Discussion

By synthesizing existing and de novo models of neuromuscular function and bioenergetics, the work presented here significantly advances our ability to investigate the relationships between individual events in the pathway of voluntary torque production and estimate their relative impact on in vivo function. Several computational models have been developed that provide unique insights into the function of individual components of neuromuscular function [3], [4], [7], [20]; however, few modeling studies have attempted to integrate across such a wide range of physiological functions. This level of integration is necessary to represent the interrelated nature of the physiological events involved in voluntary torque generation. The fidelity of model simulations with experimental data over a range of conditions demonstrates the utility of this model in future applications that might provide a deeper understanding of the relative effect of failure or enhancement at multiple points in the pathway of voluntary torque production. A particularly useful feature of the present model is the manner in which the output of individual components corresponding to MU activity, intracellular bioenergetics and joint torque (2, 4a, and 5 in Figure 2 respectively) can be compared with relevant in vivo data. Because overall model behavior depends on the integrated function of these components, manipulation of a single component within the model allows for exploration of the impact of that component on overall behavior.

Although the bioenergetics component of the model is its most novel aspect, its synthesis with other components of neuromuscular function allow us to apply the present model to a range of questions, not just those related to intracellular metabolism. The formerly established, but newly combined models of neural excitation, muscle activation, muscle contraction, and joint torque production, based on the work of Fuglevand et al [11], [42], He et al. [21], van Soest et al. [18], and Manganaris et al [19] accurately predicted the magnitude and kinetics of torque production, as demonstrated collectively in Figure 4 (a, b, and c). The combined function of these components results in muscle forces and joint torque estimates that can be readily compared with in vivo experimental observation. Adjustment of model parameters related to muscle fiber type distribution, tendon stiffness, and joint architecture (moment arm length and distension with force) could yield useful data related to the pathophysiology of disease processes or the effects of old age. Similarly, questions related to neural (dys)function in clinical populations might be addressed using the present model. Because alterations in neural function that accompany pathologies such as stroke or multiple sclerosis are also associated with changes observed at the muscle and joints, determining the etiology of reduced voluntary torque capacity is very complicated. The present model might be used to discriminate between multiple factors that impact voluntary joint torque production in these populations and by doing so, identify targets for intervention that most effectively promote improved neuromuscular function.

Valuable information was gleaned during the process of formulating the model functions and determining parameter values to create the modules presented here. While parameters describing physiological behavior were based on a combination of literature values and our own experimental results, it is important to note that most model inputs were adjusted to ensure realistic predictions by each module or component. For example, adjustment of bioenergetic functions within the model to ensure accurate predictions of pH and phosphate metabolites provided a novel insight: The value θ is a coefficient that relates to the amount of H^+^ produced or consumed in the creatine kinase reaction, and θ varies with cytosolic pH. Using values reported by Walter et al [46] caused an underestimate of alkalosis during contraction. We found that buffering capacity and θ had the greatest impact on overall predictions of pH during contraction. While our final parameter value was within the range of values observed experimentally, it was necessary to increase the value θ by 40% relative to that reported by Walter et al [46]. Our model suggests therefore, that inherent buffering capacity and the breakdown of PCr likely have the greatest impact on intracellular pH during brief, isometric contractions. This prediction agrees with experimental observations [52] but allows for the individual assessment of inherent buffering capacity in ways that are not possible in vivo. Changes to buffering capacity in vivo are accompanied by a host of other intracellular metabolic adaptations that complicate assessment of its singular impact on intracellular bioenergetic function.

While the present model formulation successfully predicts the neural, contractile, and bioenergetic responses to voluntary torque generation during relatively short contractions, it lacks any consideration for the maintenance of bioenergetic homeostasis or the consequence of metabolic changes on force production capacity. Although this limitation is unlikely to impact predictions of maximal torque during brief contractions, prolonged contractions will cause intracellular metabolic alterations that reduce force generating capacity [53]. Acidosis and increased [Pi] are well known mechanisms of muscle fatigue, due to their impact on contractile protein function, both in vitro [54], [55] and in vivo [56]. When muscle activation ceases, the present model will not predict recovery of metabolic homeostasis (eg: proton efflux from the cytosol, or resynthesis, of PCr) limiting its application to simulations involving intermittent, repeated contractions. Adding these features related to metabolic function would increase the utility of the present model by allowing for adjustment of force output in response to a changing metabolic milieu, and therefore provide realistic predictions of changes in joint torque production during prolonged, or repeated intermittent contractions. In the same way, the effects on torque output due to adjustments in MU recruitment and firing rate patterns that occur in vivo in response to metabolic feedback from the muscle to the nervous system also could be captured and examined using the comprehensive approach presented here. It should be noted that the present model has been evaluated only under isometric conditions. While our results correspond very well to a wealth of in vivo studies of neuromuscular function, in the future the model could be extended to simulate shortening and lengthening muscle actions.

It should be noted that, while the model contains functions that accurately describe the determinants of pH and [H_2_PO_4_^−^], it does not include explicit functions related to the kinetics of oxidative phosphorylation during activation, nor the role oxidative phosphorylation plays in re-establishing [PCr] following contraction. The generation of H^+^ from oxidative phosphorylation is negligible compared with the amounts produced or consumed through glycolysis or the creatine-kinase reaction, but oxidative phosphorylation plays a critical role in synthesizing ATP and maintaining [PCr] during prolonged muscle activation. Both the relatively slow onset kinetics of oxidative phoshphorylation and the very good agreement between the simulated and experimental data (Figures 5, 6, 7) suggest that the lack of an oxidative phosphorylation function in the present model does not limit its utility during the relatively brief (12-s) simulated contractions used here. However, future applications of the present model to the study of neuromuscular function during longer contraction protocols will likely require incorporation of oxidative metabolism as a model component. A model of oxidative ATP production could provide a novel approach to estimating the maintenance of cellular homeostasis during intermittent and submaximal contractions, in addition to simulating the recovery of intracellular metabolite concentrations to resting levels after contractions cease. Such a model would be useful in addressing questions related to cellular energetics and the impact of specific metabolites on muscle torque production during fatigue.

The novel contributions of the work presented here are twofold: 1) a single, comprehensive model that employs a unique, modular structure capable of predicting the neuromuscular response to a variety of contractile tasks; and 2) the integrated components within the model that allow for prediction of, and interrelationship among multiple physiological responses. The model's agreement with experimentally-derived, in vivo data, across a range of contraction intensities, highlights its utility as an adaptable tool for simulating neural, contractile and metabolic responses to a variety of conditions. Specifically, future studies might be directed at dissecting the roles of interrelated components of neuromuscular activation and bioenergetics in muscle weakness due to pathology or age, or during repetitive, fatiguing skeletal muscle contractions. For example, neurological disorders such as multiple sclerosis affect multiple aspects of neuromuscular function whose relative impact on torque producing capacity might be better estimated using the present model. Similarly, the aging process promotes systemic changes in neuromuscular function whose individual contributions to age-related declines in physical dysfunction are frequently debated. Our model provides a unique, theoretical foundation upon which to estimate the relative impact of changes at one or many points in the pathway of voluntary joint torque production and inform these debates.

## Supporting Information

Appendix S1 **Activation, Force Development and Torque Generation.**
(DOC)

Appendix S2 **Formulation of the Bioenergetic Model.**
(DOC)
