# Supplementary material for: A Computational Model of Torque Generation: Neural, Contractile, Metabolic and Musculoskeletal Components
Source: PLoS One. 2013 Feb 6;8(2):e56013. doi: 10.1371/journal.pone.0056013 (PMC3566067; doi:10.1371/journal.pone.0056013)
Supplement: Appendix S1 — Activation, Force Development and Torque Generation. (DOC) [file pone.0056013.s001.doc]

## Appendix 1: Activation, Force Development and Torque Generation

The model parameters were sourced from experimental, and published values. The model was constructed based on the following scheme. Roman numerals correspond to model steps illustrated in Figure 2:

1. An error function evaluates the difference between current torque (T*_c_*) and a pre-defined target torque (T*_t_*); Equation 1. Values for T*_t_* are calculated prior to running the model, and T*_c_* is calculated as show in equation 37.
   1. *Stimulation (S) to model adjusted*

$T_{diff}=T_{c}-T_{t}$ (1)

$\uparrow S=S+(RT_{diff}\cdot(1-S))$ (2)

$\downarrow S=S-(RT_{diff})^{r}\cdot S$ (3)

Where T*_diff_* is the absolute difference between the current and target torque values. *R*T*_diff_* is T*_diff_* expressed relative to the expected maximum torque generating capacity of the model. The exponent *r* moderates the rate of decreasing stimulation. Informal optimization for the most realistic stimulation kinetics resulted in a value of 0.7 for *r.*

1. Recruitment model determines the firing rate *(FR)* within specified range (minimum: *MFR*; peak: *PFR*) for each motor neuron (*m*: 1-120) after recruitment threshold (*muRT*) is met.

Current firing rate (*FR*) is the product of gain (*G*) and the difference between the current stimulation level (*S*) and the recruitment threshold of motor neuron *m* (*mnRT_m_*) when *S* exceeds *mnRT_m_*.

$FR=G\cdot(S-(mnRT_{m}))+MFR$ (4)

*a* is a coefficient whose value is determined by the range of desired activation thresholds (*A_range_*) and the total number of simulated motor neurons (*MN_num_*).

$a=\log(A_{range})/MN_{num}$ (5)

The recruitment threshold of the last-recruited *MN* (*RT_last_*) is a function of *a* and the index of the last *MN* (*MN_num_*).

$RT_{last}=\exp(a\cdot MN_{num})$ (6)

The recruitment of each motor neuron (*m*) follows the same function.

$mnRT_{m}=\exp(a\cdot m)$ (7)

Peak firing rate for the *m*th motor neuron is determined by subtracting the product of the range of desired firing rates (*FR_range_*) and the recruitment threshold of of the *m*th motor neuron (expressed relative to the highest recruitment threshold of the pool) from the desired peak firing rate (*PFR*).

$mnPFR_{m}=PFR-(FR_{range}\cdot(mnRT/RT_{last}))$ (8)

Where *G* = 2, the gain for the activation signal; *A_range_* = 30, the desired range of activation thresholds; *FR_range_* = 10, the desired range of peak firing rates; *MFR* = 8, the minimum firing rate in Hz; *PFR* = 56, the highest observed firing rate; and *MN_num_* = 120, the total number of motor neurons.

1. Current firing rate (*FR*) is used to calculate the inter-pulse interval (*IPI*), which is compared with the calcium transient duration (*Ca_on_* = 0.023s). At each time step during the simulation, a counter is initiated for each active motor neuron, tracking its IPI. Throughout the simulation, the active counter for reach *MN* is compared with *Ca_on_*. When the counter is less than *Ca_on_*, *Stim* = “1”. When the counter is greater than *Ca_on_*, but less than *IPI*, *Stim =* “0”. When the counter is = to *IPI*, the counter resets to zero. Activation kinetics are determined by the following equations:

$\mathop{act}\limits^{\bullet} =\left(Stim-act\right)\cdot\left(rc_{1}\cdot Stim+rc_{2}\right)$ (9)

$rc_{2}=1/t_{deact}$ (10)

$rc_{1}=1/t_{act}-rc_{2}$ (11)

where *t_act_* is the activation time constant, which has values that were distributed linearly across *MNs* in a range between 0.039 and 0.06: $t_{act(m)}=0.06-(0.35\cdot(m/MN_{num}))$). These values represent the activation time constant. Deactivation time constants (*t_deact_* ) were also distributed linearly by *MN* and have values between 0.064 – 0.092: $t_{deact(m)}=0.092-(0.3\cdot(m/MN_{num}))$.

1. Vector equations determine rates of change for state variables meant to represent contractile dynamics: The muscle model consists of a contractile element (*ce*) and series elastic element (*see*). The behaviors of these elements depended on their respective lengths (*lce,* and *sel* respectively) and their sum always equaled the length of the myotendenous unit (*lmt*) in order to simulate an isometric contraction.

Because simulated contractions are isometric, change in *lmt* is equal to zero.

$\mathop{lmt}\limits^{\bullet} =0$ (isometric) (12)

*c*_0_ is a coefficient defined by the width of the *force-length* relationship and is used to moderate isometric force generating capacity of the *ce* (*f_iso_*) depending on current *lce* with respect to the optimum force-generating capacity length *lce_opt_*.

$c_{0}={}^{-}1/\omega$ (13)

$f_{iso}=c_{0}\cdot(lce/lce_{opt})^{2}-2\cdot c_{0}\cdot(lce/lce_{opt})+c_{0}+1$ (14)

$f_{act}=act^{0.3}$ (15)

Change in *lce* depends on current activation level (*f_act_*) *f_iso_*, and maximum shortening velocity, defined by *arel* and *brel*. These are modified Hill coefficients according to van Soest et al, 1993 [18]. The following equation refers specifically to concentric contractions.

$\mathop{lce}\limits^{\bullet} ={}^{-}1\cdot f_{act}\cdot lce_{opt}\cdot((((f_{iso}+arel)\cdot brel)/(fce/(f_{\max}\cdot act)+arel))-brel)$ (16)

Pennation angle (*pen*) is calculated based on current *lce*.

$pen=(180/\pi)\cdot(a\sin((lce_{opt}\cdot\sin((\pi/180)\cdot pen_{0}))/lce))$ (17)

The current values for *pen* and *lce* are used to determine *sel*.

$sel=lmt-lce\cdot\cos(pen\cdot\pi/180)-l_{slack}$ (18)

The *see* is modeled as a non-linear spring whose force (*fsee*) is based on *sel* and a spring constant *ksee*.

$fsee=ksee\cdot sel^{2}$ (19)

Finally, force in the *ce* (*fce*) must equal that in the *see* while accounting for pennation angle.

$fce=fsee/\cos(pen\cdot\pi/180)$ (20)

Where *ω* = 0.56 (179) and *lce_opt_* = 0.058; *arel* is between 0.2 - 0.14 and *brel* is between 2.4 - 2.52. These values correspond to a and b coefficients for a typical Hill muscle model respectively and are distributed linearly, depending on *MN num*: $arel_{m}=0.2-(0.3\cdot(m/MN_{num}))$, $brel_{m}=2.4+(0.05\cdot(m/MN_{num}))$.

1. Metabolic dynamics include inorganic phosphate (*Pi*), activation of glycolysis (*Gact*) and glycolytic rate (*L*). Hydrogen production from glycolysis (*H*) is offset by consumption of proton during the creatine kinase reaction (*H_1_*). Net proton production or consumption is buffered (*H_3_*) and used to calculate the current cytosolic pH (equation 29).
2. Metabolic perturbation in response to activation

The activation level for metabolic dynamics (*act_rel_*) is expressed relative to the maximum rate of inorganic phosphate (*Pi*) accumulation observed in vivo. The foundation for this equation is described more fully in Appendix B (2), which describes the relationship between activation (*act_rel_*) and rate of PCr depletion. Because PCr and Pi are reciprocal and stoichometrically equivalent, the equations are identical but for the sign of *act_rel_*.

$act_{rel}=(0.0045+({}^{-}0.0161\cdot(act\cdot100)+4.19e^{-5}\cdot(act\cdot100)^{2}))/1.189$ (21)

During muscle model activation, *Pi* accumulates at a rate depending on *act_rel_*, the duration of the contraction (*Ct*) and coefficients defining the sigmoidal relationship between contraction duration and *Pi* concentration (*Pi_a_, Pi_b_, Pi_x_*).

$\uparrow Pi=Pi_{a}/1+(\exp(({}^{-}Ct+Pi_{x})/Pi_{b})^{2}/(Pi_{b}\cdot\exp({}^{-}Ct+Pi_{x}/Pi_{b})\cdot{}^{-}act_{rel})$ (22)

A model of glycolytic rate (*L*) depends on the activation of glycolysis and current *Pi* concentration. The activation of *L* is determined from rate constants determining activation kinetics of *L* similar to *ce* activation, both of which are dictated by *Stim*.

$Grc_{2}=1/GT_{dact}$ (23)

$Grc_{1}=1/\left(GT_{act}-Grc_{2}\right)$ (24)

$\mathop{Gact}\limits^{\bullet} =(Stim-Gact)\cdot(Grc_{1}\cdot Stim+Grc_{2})$ (25)

*Pi* was assumed to influence *L* through Michaelis Menten kinetics.

$\mathop{L}\limits^{\bullet} =\mathop{Gact}\limits^{\bullet} \cdot((L_{o}\cdot(Pi-Pi_{rest}))/(K_{m}-Pi_{rest})+(Pi-Pi_{rest}))$ (26)

Protons (*H*) generated from *L* were based on the assumption that 3 glycolytic ATP are generated per proton produced through anaerobic glycolysis.

$\mathop{H}\limits^{\bullet} =\mathop{L}\limits^{\bullet} /1.5$ (27)

Total protons produced must be balanced by those consumed in the creatine-kinase reaction. This is the product of *θ* and the breakdown of phosphocreatine, which is stoichiometrically equivalent to the amount of *Pi* generated.

$\mathop{H}\limits^{\bullet} _{1}=\mathop{H}\limits^{\bullet} _{0}-(\theta\cdot Pi)$ (28)

Finally, the total change in proton load (*H_2_*) is divided by the total cytosolic buffering capacity (*β_total_*) to determine the observed change in proton concentration (*H_3_*).

$\mathop{H}\limits^{\bullet} _{3}=\mathop{H}\limits^{\bullet} _{2}/\beta_{total}$ (29)

*GT_act_* = 0.2, the time constant for activation of glycolysis. *L_0_* is the maximum rate of glycolysis and is limited to a ~4-fold range between 0.48 - 1.92 mM. Values in this range were distributed in an exponential fashion, per *MN*, similar to *mnRT* ($Lo=\exp(bGL\cdot m)$, where $bGL=\log(4)/MN_{num}$); *Pi_rest_* = 3.94 and is the resting Pi concentration in mM; *Km* = 15 and is the concentration in mM of Pi at which glycogen phosphorylase reaches half its full activation. Coefficients for proton consumption in the creatine kinase reaction (*θ*) and oxidative phosphorylation (M) were set at 0.357 and 0.14 respectively at the onset of exercise, but varied during the simulation, according to pH [42].

1. Vector equations determine state variables for current time point (t). Variables reflecting the “intracellular state” for pH, diprotonated phosphate (H_2_PO_4-_) buffering capacity of inorganic phosphate (B_Pi_) and phosphocreatine (PCr) for each MN(m) are then updated:

$pH={}^{-}\log_{10}(H_{3})+7$ (30)

$H_{2}PO_{4-}=Pi/(1+10^{(pH-6.75)})$ (31)

(32)

$PCr=42.5-Pi$ (33)

1. Metabolic and torque values are combined for all motor neurons/muscle model pairs (motor units), to calculate the sum total of modeled behavior at each time point. These are the representative output for each respective variable and are compared directly with experimental data.

The weighted sum of metabolic variables are combined to reflect the value for each variable at each time point (*t*) for all motor units.

$MetT=\sum_{m=1}^{MU_{num}} (Met_{m}\cdot f_{\max m})/\sum_{m=1}^{MU_{num}} f_{\max m}$ (34)

Where values of *f*_max_*_m_* were distributed exponentially across a ~100-fold range (0.56 - 54.5) similar to *MN_RT_ (*$f_{\max m}=\exp(b\cdot m)$where $b=\log(100)$)

1. Force values for motor units are taken from *fsee* and summed linearly to estimate force at the tendon (*F_t_*) which is then used to calculate moment arm length (*L_ma_*) and current torque (T*_c_*).

$F_{t}=\sum_{m=1}^{MU_{num}} fsee_{(m)}$ (35)

$L_{ma}=L_{ma0}+((L_{ma0}\cdot L_{maR})/(F_{\max}/F_{t}))$ (36)

Finally, the newly calculated T*_c_* is compared with the designated T*_t_* and the preceding steps are repeated.

$T_{c}=F_{t}\cdot L_{ma}$ (37)

Where *L_ma_*_0_ is the resting moment arm length (0.027m), *L_maR_* is the potential range of increased moment arm length (0.249) and *F*_max_ is the maximum force possible at the tendon:

$\sum_{m=1}^{MU_{num}} f_{\max m}$= 1433.4 N (38)
