# Supplementary material for: A Computational Model of Torque Generation: Neural, Contractile, Metabolic and Musculoskeletal Components
Source: PLoS One. 2013 Feb 6;8(2):e56013. doi: 10.1371/journal.pone.0056013 (PMC3566067; doi:10.1371/journal.pone.0056013)
Supplement: Appendix S2 — Formulation of the Bioenergetic Model. (DOC) [file pone.0056013.s002.doc]

**Appendix 2: Formulation of the Bioenergetic Model**

A key feature of the integrated neuromuscular model (Appendix A) is the bioenergetics component (Step Va, equations 21-33). During activation, functions simulated the metabolic perturbation observed in vivo. A detailed description of the procedures followed to derive those functions follows below:

The accumulation of inorganic phosphate (Pi) and depletion of phosphocreatine (PCr) during activation are based on maximal 12-s contractions. The relationship between [Pi] and contraction time (*Ct*) was described by a sigmoidal function (*Pi_a_, Pi_b_, Pi_x_*), selected for its goodness of fit with these experimental data. Our in vivo observations were augmented by a theoretical data point at 60s according to Lanza et al ^30,37^ in order to more completely describe the relationship between *Ct* and [Pi]. The first derivative of this function determines the rate of change of [Pi] in the bioenergetics model.

$Pi=Pi_{a}/1+\exp(({}^{-}Ct+Pi_{x})/Pi_{b})^{2}/(Pi_{b}\cdot\exp({}^{-}Ct+Pi_{x}/Pi_{b}))$ (1)

The relationship describing the initial rate of PCr depletion as a quadradic function of activation level (*act*) was used to scale changes in Pi predicted from equation 1. The first derivative of this function is expressed relative to the maximum rate of PCr depletion (*Dep_max_* = -1.189) to scale the rate of change in activation (*act_rel_*).

$act_{rel}=(0.0045+({}^{-}0.0161\cdot(act\cdot100)+4.19e^{-5}\cdot(act\cdot100)^{2}))/Dep_{\max}$ (2)
